# INS, OUTS, AND UNINTENDED CONSEQUENCES OF THE CMS NURSING HOME QUALITY MEASURES

**DOI:** 10.1093/geroni/igz038.556

**Published:** 2019-11-08

**Authors:** Heather Davila, Tetyana P Shippee, Young Shin Park, Daniel Brauner, R Tamara Konetzka

**Affiliations:** 1 VA Boston Healthcare System, Boston, Massachusetts, United States; 2 University of Minnesota, Minneapolis, Minnesota, United States; 3 University of Chicago, Chicago, Illinois, United States

## Abstract

Ongoing concerns about the quality of care provided to nursing home (NH) residents have led the federal government to develop quality measures (QMs) for NHs. Many of these QMs are included in the NH 5-star ratings and reported online via Nursing Home Compare. However, we know little about how NH providers view the QMs, challenges they experience in addressing the measures, and strategies they use to achieve better scores. As part of a broader mixed-methods study to understand how NHs are responding to the 5-star ratings, we conducted interviews with NH personnel (n=110) and observed organizational processes in 12 NHs in three states. We also interviewed policy and industry leaders (n=34) to gain their perspectives. Interviews focused on perceptions of the 5-star ratings, organizational strategies to improve 5-star scores, experiences with the survey/regulatory process, and perceptions and responses to individual QMs. Key themes show that a) NH providers view the QMs as important indicators of quality, but there is variability across indicators; b) providers face challenges related to measurement and definitions for certain QMs (e.g., pain, restraints); and c) there are potentially conflicting goals, where some QMs aim to promote safety at the expense of resident autonomy and quality of life and vice versa. This work provides organizational context to the 5-star measures and the balancing act providers engage in to assess and improve their scores. The findings also identify several potentially unintended consequences related to the QMs, which can adversely affect residents, particularly those with more complex care needs.

